# Morphological and Spatial Diversity of the Discal Spot on the Hindwings of Nymphalid Butterflies: Revision of the Nymphalid Groundplan

**DOI:** 10.3390/insects11100654

**Published:** 2020-09-23

**Authors:** Joji M. Otaki

**Affiliations:** The BCPH Unit of Molecular Physiology, Department of Chemistry, Biology and Marine Science, Faculty of Science, University of the Ryukyus, Okinawa 903-0213, Japan; otaki@sci.u-ryukyu.ac.jp; Tel.: +81-98-895-8557

**Keywords:** butterfly wing, central symmetry system, color pattern, color pattern element, discal spot, nymphalid groundplan, wing development

## Abstract

**Simple Summary:**

Butterfly wing color patterns are diverse, but they can be understood as modifications of the common scheme called the nymphalid groundplan. The discal spot is relatively small, but it is one of the important components of the nymphalid groundplan. Using many hindwing specimens of the family Nymphalidae, the morphological and spatial diversity of the discal spot was studied. The discal spot is expressed as a small or narrow spot, a pair of parallel bands, a diamond or oval structure, a large dark spot, a few fragmented spots, or a white structure. The discal spot is always located in a central portion of the wing defined by the wing veins, and this portion is sandwiched by a pair of bands of the central symmetry system, another important component of the nymphalid groundplan. On the basis of these results, the present study revises the nymphalid groundplan in minor points; the discal spot is an independent and diverse miniature symmetry system nested within the central symmetry system. Due to the involvement of wing veins to define the locations of the discal spot, the present study suggests the possible developmental dynamics of butterfly color pattern formation that produces color pattern diversity.

**Abstract:**

Diverse butterfly wing color patterns are understood through the nymphalid groundplan, which mainly consists of central, border, and basal symmetry systems and a discal spot. However, the status of the discal spot remains unexplored. Here, the morphological and spatial diversity of the discal spot was studied in nymphalid hindwings. The discal spot is expressed as a small or narrow spot, a pair of parallel bands, a diamond or oval structure, a large dark spot, a few fragmented spots, or a white structure. In some cases, the discal spot is morphologically similar to and integrated with the central symmetry system (CSS). The discal spot is always located in a distal portion of the discal cell defined by the wing veins, which is sandwiched by the distal and proximal bands of the CSS (dBC and pBC) and is rarely occupied by border ocelli. The CSS occasionally has the central band (cBC), which differs from the discal spot. These results suggest that the discal spot is an independent and diverse miniature symmetry system nested within the CSS and that the locations of the discal spot and the CSS are determined by the wing veins at the early stage of wing development.

## 1. Introduction

Insects have a large variety of morphological structures and color patterns. Among them, color patterns on butterfly wings are very diverse. They are so diverse that they have attracted the attention of biologists who are interested in pattern formation and morphogenesis. Some researchers have searched for a unified understanding of the diversity of butterfly wing color patterns. As early as 1924, Spemann and Mangold (1924) [1,2] formulated a basic concept of organizers in experimental embryology, and Schwanwitsch (1924) [3] and Süffert (1927) [4] independently proposed that diverse color patterns are derivable from an archetypical overall color pattern called the nymphalid groundplan (NGP). A modern version of the nymphalid groundplan was proposed by Nijhout (1991, 2001) [5,6] and was later modified in minor points and expanded by Otaki (2012, 2018) [7,8]. The nymphalid groundplan is a conceptual framework for identifying anatomically homologous color patterns among different species. Thus, diverse butterfly color patterns can be understood as modifications of the nymphalid groundplan in color, shape, size, and location of color pattern components called color pattern elements (or simply elements) regardless of bands or spots.

**Figure 1:**
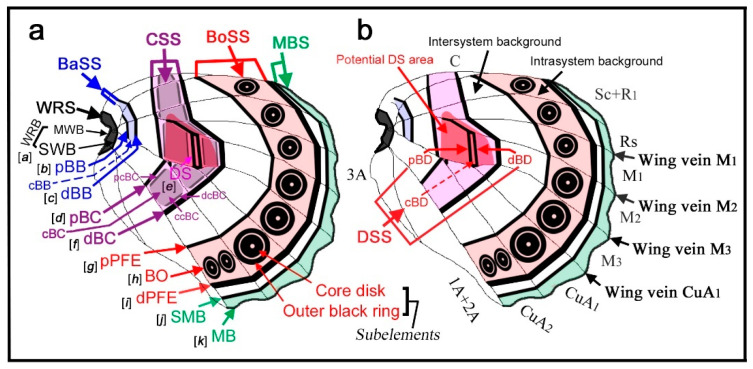
The nymphalid groundplan (NGP) of the hindwing. The basic scheme and nomenclature are based on Nijhout (1991) [5] and Otaki (2012) [7]. This illustration incorporates the results of previous studies [7,8] and the present study. All symmetry systems are considered to have a core element and a pair of paracore elements according to the core–paracore rule [7,8], although core elements are not always depicted in this illustration for simplicity. The wing root band system is considered a half symmetry system, and accordingly, it is considered to have a core element and a paracore element similar to the marginal band system. In this illustration, the basal symmetry system (BaSS) is drawn, but discalis II (DII) is not, because they are not differentiated in the literature. However, they may be different entities. Thus, the illustration of the BaSS is tentative. (**a**) The nymphalid groundplan with annotations of systems, elements, and subelements. The field of each system is color-coded. Both nomenclature-based abbreviations (two to four letters) and alphabetical assignments (in brackets from a to k) are shown. Abbreviations: BaSS: basal symmetry system; CSS: central symmetry system; BoSS: border symmetry system; MBS: marginal band system; WRS: wing root band system; BO: border ocellus (eyespot); dPFE: distal parafocal element; pPFE: proximal parafocal element; dBC: distal band of the central symmetry system; pBC: proximal band of the central symmetry system; cBC: central band of the central symmetry system; dcBC: distal band of cBC; pcBC: proximal band of cBC; ccBC: central band of cBC; dBB: distal band of the basal symmetry system; pBB: proximal band of the basal symmetry system; cBB: central band of the basal symmetry system (not depicted in the wing illustration); MB: marginal band; SMB: submarginal band; MWB: marginal wing root band; SWB: submarginal wing root band; WRB: wing root band; DS: discal spot. (**b**) Nomenclature of wing veins and wing compartments [11], the vein-dependent definition of the potential DS area, and the discal symmetry system. Here, cBC is entirely omitted to clarify the discal symmetry system. The proximal part of the wing compartment M_2_ is either open or closed by the discal cross vein. The closed compartment is called the discal cell (or simply the cell). The potential DS area, located in the discal cell (indicated in red), was determined as the most frequent area for the presence of the discal spot as a result of the present study (see Section 3.3). In this illustration, the discal spot is shown as a pair of parallel bands on the discal cross vein and a band on the most proximal side of the wing vein M_2_. Two different types of background (intrasystem background and intersystem background) are indicated. The background area within a system field is the intrasystem background, whereas the background area between two systems is the intersystem background. Abbreviations: DSS: discal symmetry system; dBD: distal band of the discal symmetry system; pBD: proximal band of the discal symmetry system; cBD: central band of the discal symmetry system (not depicted in the wing illustration).

In the nymphalid groundplan of the hindwing (Figure 1a,b) (but also of the forewing), there are three major symmetry systems: the central symmetry system (CSS) at the center, the border symmetry system (BoSS) at the distal side, and the basal symmetry system (BaSS) at the proximal side. They often transverse the entire wing surface from the anterior (costal) to posterior (hind) margins as bands or serial spots. Each system is composed of collections of elements. In this sense, the nymphalid groundplan can be viewed as placing elements on a plain background. However, this way of thinking about the nymphalid groundplan is somewhat misleading in the following two points. First, it has been thought that the background itself may be colored [5]. When the background is partially colored, a bold area with an ambiguous boundary occurs (“bold background pattern” [5]). Additionally, there is a possibility that dark background coloration is produced by an extreme expansion of a single element, resulting in a semi-element [9,10]. Second, there appear to be several color pattern rules that specify how elements should be placed and constructed [8]. Without these rules, a detailed understanding of the nymphalid groundplan would be impossible.

Some minor modifications of the nymphalid groundplan have been introduced [7,8] since Nijhout (1991) [5]. First, the status of the parafocal element (PFE) has been established as a part of the border symmetry system [12,13,14,15,16,17]. Second, the proximal parafocal element (pPFE) in addition to the distal parafocal element (dPFE) has been identified [7,12]. Third, an introduction of the proximal submarginal band (pSMB) that was tentatively proposed was not correct, and it was soon identified as a part of the distal band of the central symmetry system (dBC) [7,12,15]. The reason for this confusion is that dBC often differentiates into double or multiple bands. Accordingly, the doubled dBC has been incorporated into the image of the nymphalid groundplan of Otaki (2012) [7]. Fourth, the submarginal band (SMB) has been identified as a part of the marginal band system [7,16]. Fifth, the wing root band system has been reintroduced [7]. Finally, but most importantly, it has been proposed that a unit of a symmetry system is a core element at the center and a pair of paracore elements at both the distal and proximal sides of the core element [7,8]. According to this core–paracore rule, not only the border symmetry system but also the central and basal symmetry systems are supposed to have a core element at the center. With these minor points considered, most elemental placements in the nymphalid groundplan have likely been valid.

Notably, the discal spot (DS) is present at the physical center of a wing, which may be considered a spot of bean-like or reniform shape. The discal spot is sandwiched by the distal and proximal bands of the central symmetry system (dBC and pBC) in the nymphalid groundplan. That is, the discal spot is nested within the central symmetry system. In addition, the location of the discal spot is exceptional. In the nymphalid groundplan, the discal spot is on the discal cross vein, which closes the central wing compartment called the discal cell (or simply the cell). The discal spot is the sole element (excluding the vein-dependent patterns and other nonelemental patterns) that is present on the wing vein in the nymphalid groundplan. Other elements are located between wing veins in a compartment, at least in nymphalid butterflies, and their centers are most often located at the midline of a compartment, which is a generalization of the midline rule for eyespot center location [8].

Most likely partly because of these peculiar characteristics, the status of the discal spot is somewhat ambiguous in the literature despite its prime importance in identifying elements based on the nymphalid groundplan. It appears that Schwanwitsch (1924) [3], Süffert (1927) [4], and Nijhout (1991) [5] all recognized the discal spot as a kind of symmetry system, because it has been well known that eyespot-like discal spots are prominent in many Saturniidae moths and some Riodinidae butterflies. To confirm this idea, Otaki (2012) [7] showed an example of a circular discal spot using species of Nymphalidae, suggesting that the discal spot may be an independent symmetry system also in Nymphalidae. However, the discal spot was yet considered a part of the central symmetry system in Otaki (2012) [7], despite that it was rather treated as an independent entity in Nijhout (1991) [5]. 

**Figure 2:**
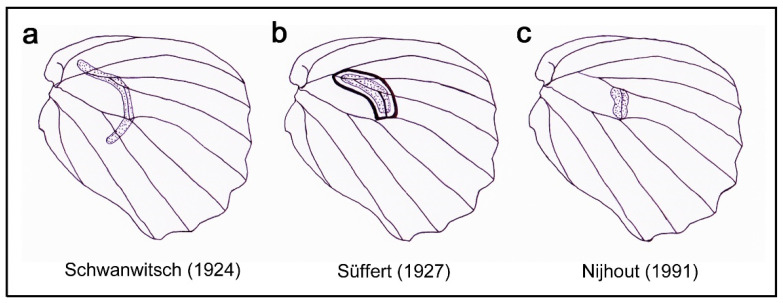
The discal spot morphology in three illustrations of the nymphalid groundplan. (**a**) Schwanwitsch (1924) [3]. (**b**) Süffert (1927) [4]. (**c**) Nijhout (1991) [5,6].

Inexplicably, the status of the discal spot has not been seriously examined in their papers. Indeed, the structure, size, and location of the discal spots in the nymphalid groundplan in Schwanwitsch (1924) [3], Süffert (1927) [4], and Nijhout (1991) [5] are all different in the hindwing (Figure 2), although their size and location in the forewing are identical among the three. Although Nijhout (1991) [5] synthesized the previous literature regarding the nymphalid groundplan, this discrepancy has not been discussed in detail. In Schwanwitsch (1924) [3], the discal spot is a pair of parallel bands that fuse together at both anterior and posterior ends without a core element and covers the seven compartments from Sc+R_1_ to CuA_2_ (Figure 2a). Alternatively, this discal spot may be considered an extremely elongated spot. In Süffert (1927) [4], the discal spot is a pair of parallel bands that fuse at both anterior and posterior ends with a core element at the center and covers the three compartments from Rs to M_2_ (Figure 2b). Thus, this discal spot is much shorter than the previous one and is more complex, similar to a moderately elongated eyespot, suggesting that the discal spot may be qualified as a small symmetry system. In Nijhout (1991) [5], the discal spot is defined as a simple spot just on the discal cross vein, covering the single compartment (Figure 2c). Otaki (2012) [7] simply followed Nijhout (1991) in the illustration of the nymphalid groundplan without critical evaluation of actual discal spots [5]. Oddly, in Schwanwitsch (1956) [18], the discal spot of the hindwing is a simple spot, as in Nijhout (1991) [5].

Additionally, Schwanwitsch (1924, 1956) [3,18] identified not only the discal spot called discalis I (DI or D^1^) but also another one called discalis II (DII or D^2^). This nomenclature is probably because of their structural similarity and spatial proximity, but implications of their similarity in the nymphalid groundplan have been uncertain. The DII spot is treated as a part of the basal symmetry system in Süffert (1927) [4], Nijhout (1991) [5], and Otaki (2012) [7], and the present study follows the current annotation [5,7], although this may not be necessarily correct. The DII identity has been studied molecularly and shown to be a serial homolog of the discal spot; both DI and DII express *wingless*, they are positioned in parallel with each other within the same compartment (the discal cell), they have similar coloration and shape, they are equally sensitive to heparin treatment that affects *Wnt* signaling pathways, and DII exists always together with DI (although DI can be present alone) [19,20,21,22]. I speculate that DII is a distinct entity from the basal symmetry system per se. However, to incorporate these molecular results and such speculation into the nymphalid groundplan requires further studies, which is beyond the scope of the present study. The present study does not focus on this issue and simply follows a conventional view that the basal symmetry system is an independent symmetry system.

Notwithstanding these differences in recognition of the discal spot among researchers in illustrations (Figure 2) and the importance of the discal spot in the nymphalid groundplan, detailed morphological analyses of the discal spot regarding structure, size, color, and location have not been reported in nymphalid butterflies. To resolve this issue, the present study examined morphological structures and locations of the discal spot from various nymphalid butterflies. Only species of Nymphalidae were used. As a result, I introduce minor but important modifications and interpretations of the nymphalid groundplan, which will contribute to a deeper understanding of how butterfly wing color patterns are generally configured in the course of evolution and development.

## 2. Materials and Methods

The hindwings of butterflies, including their dorsal and ventral sides, were visually examined throughout the family Nymphalidae (Lepidoptera). Butterfly specimens were obtained from the author’s personal collection. The choice of specimens that are shown in figures was based on the author’s discretion, but species that likely show representative patterns to the best of the author’s knowledge were accepted for figures, provided that specimens were available for the author to do so. Since this study is to understand the nymphalid groundplan sensu stricto, only specimens of Nymphalidae were used. 

The exclusion of the forewings in this study is basically for simplicity. However, only in the hindwings did the three types of nymphalid groundplans disagree on the discal spot. Furthermore, it is likely that the forewing patterns are generally more complicated than the hindwing patterns in terms of dislocation of elements. Dislocation of the bands of the central symmetry system, i.e., “pierellization” [5,23], may not occur as often in the hindwings. Pierellization occurs because each wing compartment is an independent unit of color pattern development [24,25]. Identifications of elements and importance of pierellization can be seen in some studies [26,27,28,29]. Moreover, in the forewings, the discal spot and the central symmetry system are probably often more complicated with surrounding elements than in the hindwings. For example, the distal band of the central symmetry system (dBC) is often expressed as double or multiple bands [7].

Wings of butterfly specimens were photographed, and their images were edited using Adobe Photoshop Elements 2019 (Adobe Systems, San Jose, CA, USA). Wing images were placed in figures so that the base was to the left and the anterior (costal) margin to the top. Wing images were adjusted to similar sizes so that color pattern comparisons were made easy.

As in previous studies [3,4,5,6,7,8,26,27,28,29], identification processes for elements were based on a “homology search” between different symmetry systems in the same wing surface, between closely related species, and between different individuals of the same species. Identification of the discal spot was basically made in reference to wing veins and to the distal and proximal bands of the central symmetry system (dBC and pBC), provided that these bands were expressed. Based on the core–paracore rule for a morphogenesis unit of a symmetry system [7,8], the central band of the basal symmetry system (cBC) was recognized as an independent element in this study. Furthermore, cBC was further dissected into the distal, proximal, and central bands of cBC (pcBC, dcBC, and ccBC), when possible.

Color pattern systems and elements in the nymphalid groundplan have unique names, which are abbreviated in three or four letters (Figure 1a,b). In addition, they have alphabetical assignments from “a” to “k” (Figure 1a). The author prefers abbreviations of names to alphabetical assignments because relationships among elements are clearer by names and because additions and deletions of elements can be handled more easily in the former. Some studies [19,20,21] use the original nomenclature of Schwanwitsch (1924, 1956) [3,18].

## 3. Results

### 3.1. Simple Spot or Line on the Discal Cross Vein and Its Extension

**Figure 3:**
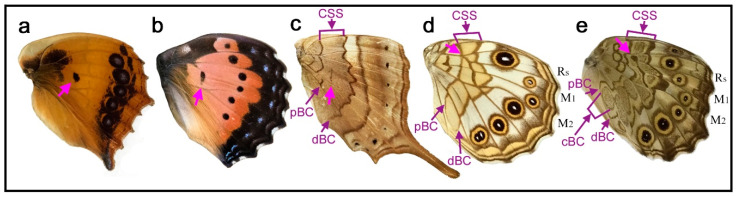
Spot or line expression of the discal spot. Pink arrows indicate the discal spot. See Figure 1 for abbreviations. (**a**) *Catacroptera cloanthe*. (**b**) *Precis octavia* (wet-season form). (**c**) *Marpesia furcula*. (**d**) *Kirinia fentoni*. The compartments Rs, M_1_, and M_2_ are indicated. (**e**) *Neope goschkevitschii*. The compartments Rs, M_1_, and M_2_ are indicated. In this species, cBC is present as an oval structure like a core disk of an eyespot.

In *Catacroptera cloanthe* (Figure 3a), the discal cross vein is not present, but the discal spot is present as an isolated single spot on the expected location where the discal cross vein is supposed to be located as an extension of the wing vein CuA_1_. This location appeared to be the most common, which may be considered the “default”, as shown in the nymphalid groundplans of Nijhout [5] and Otaki [7]. The structure, color, and size of this discal spot are also in accordance with the nymphalid groundplans of Nijhout (1991) [5] and Otaki [7]. Thus, it may be called Nijhout’s type in location, structure, color, and size. The location of the discal spot is slightly more proximal in relation to wing veins in *Precis octavia* (wet-season form) (Figure 3b), whose discal spot is a distinct spot located between the end of the wing veins CuA_1_ and CuA_2_. In these two species, the central symmetry system, which flanks the discal spot, is not present.

The discal spot is expressed as a thin line on the top of the discal cross vein in *Marpesia furcula* (Figure 3c). In this species, because of the elongated wing shape, the discal spot appears to be located relatively more proximally than that of *Catacroptera cloanthe* (Figure 3a), but its location in relation to wing veins is also the default. This finding suggests the importance of wing veins (especially the discal cross vein, which closes the compartment M_2_ to form the discal cell) in relation to the entire wing shape for the relative positions of the discal spot and likely other elements. In this species, the central symmetry system is expressed as a pair of parallel bands, the distal and proximal bands of the central symmetry system (dBC and pBC), which sandwich the discal spot. 

In *Kirinia fentoni* (Figure 3d), there is a thin discal cross vein, and the discal spot is on that wing vein as a thin line like in the previous species. However, the discal spot itself extends anteriorly on the veins, covering the most proximal sides of the three compartments M_2_, M_1_, and Rs. The discal spot of *Neope goschkevitschii* (Figure 3e) shows basically the same configuration as that of the previous species. In terms of location, the discal spot of these two species is Süffert’s type but is less elaborated in its structure than that of Süffert’s. Notably, *Neope goschkevitschii* (Figure 3e) has the central band of the central symmetry system (cBC), an oval structure similar to an eyespot core disk, flanked by dBC and pBC.

### 3.2. A Pair of Parallel Bands near the Discal Cross Vein and Its Extension

**Figure 4:**
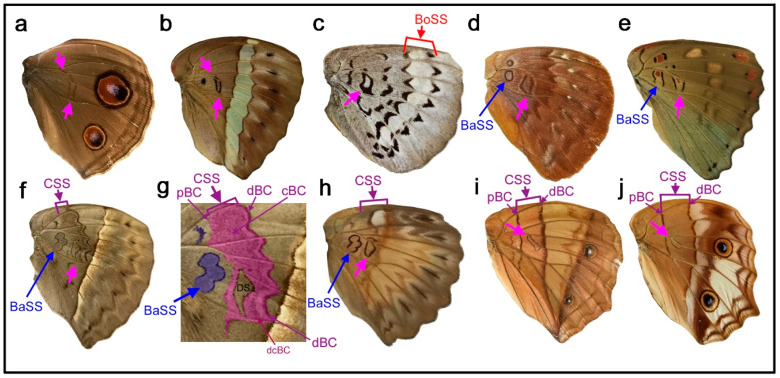
Double-band expression of the discal spot. Pink arrows indicate the discal spot. See Figure 1 for abbreviations. (**a**) *Junonia artaxia*. (**b**) *Bassarona teuta*. (**c**) *Tanaecia trigerta*. (**d**) *Euryphura achlys*. (**e**) *Euthalia lubentina*. (**f**) *Cymothoe egesta*. (**g**) The discal area of the hindwing shown in f. The central symmetry system (CSS) and the basal symmetry system (BaSS) are indicated in red-purple and blue-purple, respectively. The discal spot (DS) is also indicated. (**h**) *Cymothoe coccinata.* (**i**) *Vindula dejone*. (**j**) *Vindula sapor*.

Two species, *Junonia artaxia* (Figure 4a) and *Bassarona teuta* (Figure 4b), show discal spots at similar locations, not only on the discal cross vein (which closes the compartment M_2_) but also on the vein closing the compartment M_1_. In addition, in these two species, the discal spot is “doubled” as a pair of parallel bands, which may be called the distal and proximal bands of the discal spot (dBD and pBD). In *Tanaecia trigerta* (Figure 4c), the discal spot is clearer than in the previous two species, with a pair of parallel bands located between the ends of the wing veins CuA_1_ and CuA_2_ and extending to the proximal side of the compartment M_1_. This location is not the default. The two bands of the discal spot merge at the anterior and posterior ends, forming a diamond shape, and the enclosed area is whiter than its surrounding area. In this species, the border symmetry system lacks a core element and expresses a pair of parafocal elements (dPFE and pPFE), between which an intrasystem white area is present. Based on the homology with the intrasystem white area of the border symmetry system, the discal spot of this species suggests that it is likely an independent symmetry system despite its smallness. A discal spot of similar shape and location is present in *Euryphura achlys* (Figure 4d), in which there are two spots of the basal symmetry system (or DII) similar to the discal spot. This discal spot has a core element, although in low contrast, and in this sense, it is Süffert’s type in structure. The core element may be called the central band of the discal spot (cBD).

In *Euthalia lubentina* (Figure 4e), the location of the discal spot is slightly distal to the end of the wing vein CuA_1_. Interestingly, a dark red spot is present at the center of a pair of parallel bands of the discal spot (dBD and pBD). The dark red spot may be considered the central band (core element) of the discal spot (cBD). This dark red spot at the center of the discal spot is not specific for the distal spot in this species; other elements of different systems (i.e., the basal and border symmetry systems) also have dark red spots on the same wing surface. This homologous coloration suggests that the discal spot is an independent symmetry system despite its smallness. This discal spot may also be considered Süffert’s type in structure.

In *Cymothoe egesta* (Figure 4f,g), the doubled discal spot is present at the end of the wing vein CuA_1_ at the default location. The overall shape of the discal spot is nearly triangular. The distal band of the central symmetry system (dBC) is clearly expressed, and the proximal band (pBC) is also identifiable, although thinner. The discal spot is nested within the central symmetry system. The discal spot is surrounded by a peppered area, and this bold peppered band inside the central symmetry system but outside the discal spot is the central band of the central symmetry system (cBC). Moreover, cBC has its own distal band (dcBC). Proximal to the discal spot, a similar element is present in the same cell, which is the basal symmetry system (or DII). Importantly, the central symmetry system, the discal spot, and the basal symmetry system are all morphologically similar to one another, except for their sizes. A similar triangular discal spot and basal symmetry system (or DII) are also present in *Cymothoe coccinata* (Figure 4h). In this species, the central symmetry system is expressed weakly.

Further diversity was found in the genus *Vindula*. In *Vindula dejone* (Figure 4i) and its related species *Vindula sapor* (Figure 4j), the discal spot is doubled and located only on the wing vein closing the compartment M_1_ but not on the discal cross vein, showing another kind of spatial diversity. In these species, dBD and pBD fuse together at both the anterior and posterior ends, forming an elongated oval. A clear pair of the bands of the central symmetry system (dBC and pBC) that flank the discal spot is present in both species.

### 3.3. Large or Multiple Spots near the Discal Cross Vein and Its Extension

**Figure 5:**
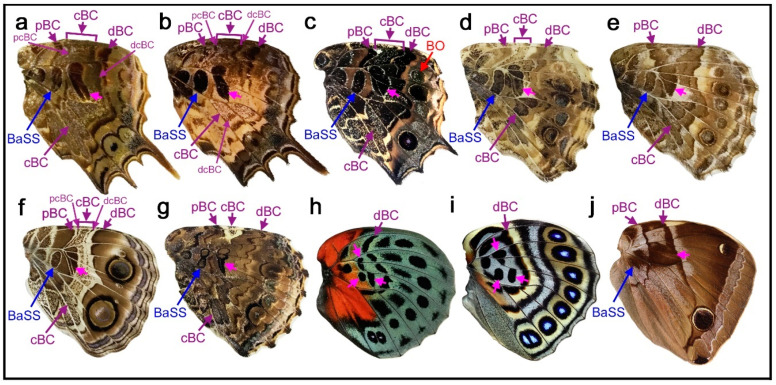
Large-spot or multiple-spot expression of the discal spot. Pink arrows indicate the discal spot. See Figure 1 for abbreviations. (**a**) *Antanartia schaeneia*. (**b**) *Antanartia delius*. (**c**) *Vanessa* (*Antanartia*) *dimorphica*. (**d**) *Vanessa* (*Cynthia*) *carye*. (**e**) *Vanessa* (*Cynthia*) *kershawi*. (**f**) *Vanessa* (*Cynthia*) *virginiensis*. (**g**) *Vanessa atalanta*. (**h**) *Agrias beatifica*. (**i**) *Agrias claudina*. (**j**) *Thaumantis odana*.

In the species discussed thus far, the discal spot is relatively small and not very conspicuous compared to the other elements on the same wing surface. However, in *Antanartia schaeneia* (Figure 5a) and *Antanartia delius* (Figure 5b), the discal spot may be recognized as one of the largest elements on the wing surface. The discal spot is a dark elongated dot and covers the proximal ends of the compartments M_2_, M_1_, and Rs, but in the compartment Rs, the discal spot is not on the wing vein and is located directly within that compartment. Since the shape of these discal spots is slightly irregular, they may be considered as fusions of a few spots. A basal symmetry system (or DII) with similar structures is also present. In these species, the distal and proximal bands of the central symmetry system (dBC and pBC) are present, between which the central band (cBC) is located as a bold band. That is, the discal spot is embedded within the cBC, and it is clearly distinguishable. Interestingly, the cBC appears to form its own distal and proximal bands, which are the distal and proximal bands of the cBC (dcBC and pcBC).

In *Vanessa* (*Antanartia*) *dimorphica* (Figure 5c), the discal spot shows a similar location to those of the previous two species, which is outlined with white scales. This discal spot is also embedded in the proximal side of the cBC. In this species, most elements, including the band of the basal symmetry system (or DII) and eyespots (border ocelli; BO) of the border symmetry system, are similar in coloration, which makes a distinction between the discal spot and the cBC (and other elements) difficult without previous knowledge of *Antanartia* species.

In *Vanessa* (*Cynthia*) *carye* (Figure 5d), the discal spot is in a similar location to that in the previous three species. Interestingly, two spots of the cBC in the compartments M_3_ and CuA_1_ in this species are dark in color, similar to the discal spot. Nevertheless, it is reasonable to state that these spots are the cBC and not the discal spot based on the knowledge of the previously discussed species. In *Vanessa* (*Cynthia*) *kershawi* (Figure 5e), the discal spot is continuous in color with pBC on the anterior side (the compartments Sc+R_1_ and C) and with the cBC on the posterior side (the compartments M_3_, CuA_1_, CuA_2_, 1A+2A, and 3A). In this species, the discal spot may be misidentified as the pBC or cBC. The correct identification of the discal spot requires knowledge of the previously discussed species. A lesson from these cases is that the location of the discal spot is likely more important than its shape and color for identification.

In *Vanessa* (*Cynthia*) *virginiensis* (Figure 5f), the dBC and pBC are thinner than those of the previous species, and the intrasystem background (i.e., white area) is relatively large, within which the cBC is identifiable as a peppered area. Furthermore, the cBC is composed of the distal, proximal, and central bands (dcBC, pcBC, and ccBC). The discal spot is identifiable at the proximal side of the cBC. In this species, the discal spot and the cBC are easily distinguishable. In *Vanessa atalanta* (Figure 5g), the discal spot is a dagger-like structure. Interestingly, the cBC on the posterior side (the compartments M_3_, CuA_1_, CuA_2_, 1A + 2A, and 3A) resembles that of *Vanessa* (*Antanartia*) *dimorphica* (Figure 5c), whereas the cBC on the anterior side (the compartments Sc+R_1_ and C) resembles that of *Vanessa* (*Cynthia*) *virginiensis* (Figure 5f). The dagger-like discal spot is different from these cBC spots. In this way, the cBC is changeable in shape, color, and size, independent of the discal spot. Therefore, the cBC is certainly a different entity from the discal spot.

In *Agrias beatifica* (Figure 5h) and *Agrias claudina* (Figure 5i), three distinct spots with similar sizes are present near the discal cross vein. The most anterior spot spans both the compartments M_1_ and Rs. Likely, they all belong to the discal spot, considering that they could fuse together if enlarged to form a type of discal spot seen in the previous *Antanartia* and *Vanessa* species.

Interestingly, in *Thaumantis odana* (male) (Figure 5j), the discal cell area in proximity to the compartments M_2_, M_1_, and Rs forms a single dark spot flanked by the dBC and pBC. The discal spots that are examined in this paper are mostly located in this area. It is likely that the discal spot of this species coincides with this common area. In reference to the discal spot of this species and other species presented in this section, I defined the potential DS area (or simply the DS area), which covers the distal portion of the discal cell and its vicinity and is flanked by the dBC and pBC (see Figure 1). I stress the importance of the potential DS area to identify the discal spot. In other words, any spots located outside this area and its proximity are not the discal spot by definition. This definition may be considered an area extension of Süffert’s type.

### 3.4. Diversity of the Discal Spot in the Genus Cethosia

**Figure 6:**
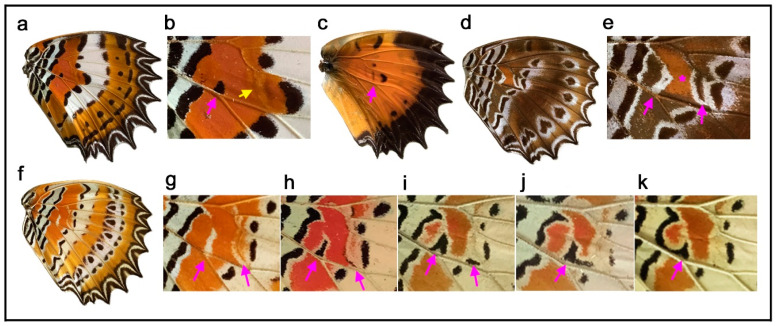
Diversity of the discal spot in the genus *Cethosia*. Pink arrows indicate the discal spot. (**a**) *Cethosia hypsea* (ventral side). (**b**) The discal area of the hindwing shown in a. A yellow arrow indicates the discal cross vein. (**c**) *Cethosia hypsea* (dorsal side). This wing is the same as that shown in a. Note the different location of the discal spot from the ventral side (a, b). (**d**) *Cethosia cydippe*. (**e**) The discal area of d. There are distal and proximal bands of the discal spots (dBD and pBD), between which the area is light brown (pink asterisk). (**f**) *Cethosia biblis*. (**g**) The discal areas of the hindwing shown in f. (**h**–**k**) The discal area of *Cethosia biblis*. These images were obtained from different individuals.

As shown in the previous sections, the discal spot is located on the discal cross vein as the default, but many nymphalid butterflies lack this vein. However, this fact may not be a reason that the location of the discal spot in relation to the wing veins is variable within the potential DS area. In the genus *Cethosia*, the discal spot is morphologically and spatially variable despite the presence of the discal cross vein. In *Cethosia hypsea* (Figure 6a,b), the discal spot is a small triangular spot located at the end of the wing vein CuA_2_ (but not at the default CuA_1_ location) on the ventral side of the hindwing. In contrast, on the dorsal side of the same wing, the discal spot is expressed as a weak narrow band on the discal cross vein at the end of the wing vein CuA_1_ (but not CuA_2_) (Figure 6c). A larger crescent spot distal to the discal spot is a spot of the distal band of the central symmetry system (dBC), considering its correspondence to the spot on the ventral side. This is a case in which different wing surfaces can express discal spots with different morphologies and locations.

In *Cethosia cydippe* (Figure 6d,e), a black band surrounded by a white area is located not only at the end of the wing vein CuA_1_ (the default location) but also at the end of the wing vein CuA_2_. The latter has only a vanishing black area at the center. These two bands are likely the dBD and pBD. The area between these two bands is light brown, suggesting the specialty of this area. This area is likely the field defined by the dBD and pBD.

In *Cethosia biblis* (Figure 6f–k), individual variation in the discal spot was observed. In the first individual, there are white bands without black areas inside both at the end of the wing veins CuA_1_ and CuA_2_ (Figure 6f,g). They are likely rudimentary (i.e., immature) bands of the discal spot (dBD and pBD). In the second individual, a triangular spot is located at the end of the wing vein CuA_2_, but there is a vanishing weak black line along the discal cross vein (Figure 6h). In the third individual, an inverted V-shaped spot is located at the end of the wing vein CuA_2_, and there is a horizontal black band near the discal cross vein, which may be the dBD or a part of the dBC (Figure 6i). In the fourth individual, these two black spots in the third individual are expressed continuously as a single entity (Figure 6j). In the fifth individual, there is an inverted V-shaped spot that fuses with the pBC, forming a larger C-shaped spot (Figure 6k).

These color patterns in the discal spot confirmed the previous findings that the discal spot is not restricted on the discal cross vein but is placed in the potential DS area and that the discal spot is structurally diverse. The discal spot may be composed of the dBD and pBD that sandwich the intrasystem background, which may be highlighted, although the cBD was not observed in *Cethosia*. These cases also indicated that discal bands tend to fuse with the bands of the central symmetry system.

### 3.5. Distinct White Structures

**Figure 7:**
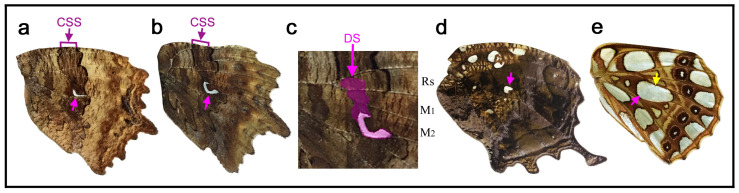
White expression of the discal spot. Pink arrows indicate the discal spot. See Figure 1 for abbreviations. (**a**) *Nymphalis vaualbum*. (**b**) *Polygonia c-album*. (**c**) The discal area of the hindwing shown in b. The discal spot (DS) is colored in pink. The compartments Rs, M_1_, and M_2_ are indicated. (**d**) *Hypna clytemnestra*. (**e**) *Issoria lathonia*. The yellow arrow indicates the discal cross vein.

A striking type of discal spot is present in *Nymphalis vaualbum* (Figure 7a). In this species, a distinct white crescent structure is conspicuous. In *Polygonia c-album* (Figure 7b,c), the white crescent structure is more distinct, as its name implies. The white area may correspond to the intrasystem white band found in *Tanaecia trigerta* above (Figure 4c). The most distal part of the white area is slightly distal to the end of the wing vein CuA_1_. The basal symmetry system (or DII) is also present. Somewhat surprisingly, the white crescent area is anteriorly extended to the “conventional” discal spot structure in this species (Figure 7c). This structure is also likely the discal spot and not the central band of the central symmetry system (cBC), in comparison to the relatively large discal spots in *Antanartia* (Figure 5a,b) and *Vanessa* (Figure 5c–g), which were referred to in defining the potential DS area (see Section 3.3).

A similar white spot is present in *Hypna clytemnestra* (Figure 7d). In this species, in addition to the discal spot, other spots near the anterior margin are also expressed as white spots. In *Issoria lathonia* (Figure 7e), pearly white spots are present all over the wing surface. One of them includes the discal cross vein at the center, which is likely the discal spot.

### 3.6. Fusion or Integration with the Bands of the Central Symmetry System

**Figure 8:**
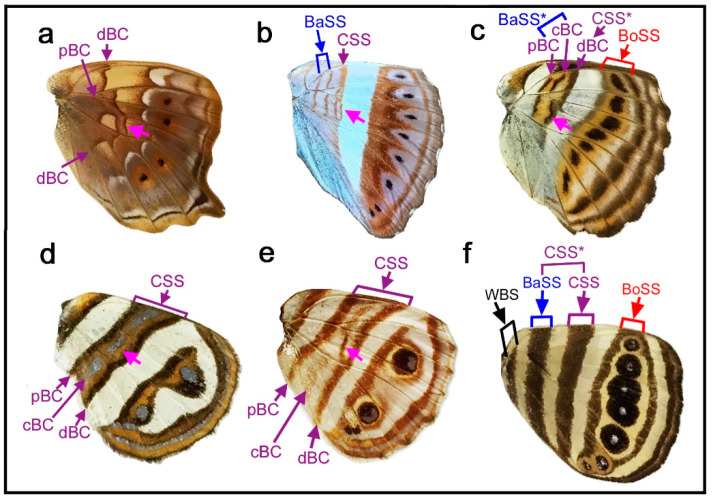
Fusion or integration of the discal spot with the central symmetry system. Pink arrows indicate the discal spot. Alternative identifications are indicated with asterisks in c and f. (**a**) *Vagrans egista*. (**b**) *Sumalia daraxa*. (**c**) *Tarattia lysanias*. (**d**) *Dynamine aerata*. (**e**) *Dynamine postverta*. (**f**) *Ragadia luzonia*.

Fusion or integration of the discal spot with the bands of the central symmetry is not rare. In *Vagrans egista* (Figure 8a), the discal cross vein is immediately sandwiched by a pair of parallel bands, which makes their identity the discal spot (dBD and pBD). Furthermore, the dBD continues toward the anterior side. The bands in the compartments M_1_ and Rs may be considered the discal spot because they are located in the potential DS area. If so, the bands in the anterior compartment Sc+R_1_ and posterior compartments CuA_1_ and CuA_2_ are the dBC, and they are morphologically indistinguishable from the bands of the discal spot (dBD and pBD). Additionally, the pBD likely merges anteriorly with the proximal band of the central symmetry system (pBC), and the pBC merges with the basal band (BB) (or DII), forming an inverted U-shaped curve. Thus, in this species, if these identifications based on the discal cross vein and the potential discal area are correct, the distal spot is integrated with the central symmetry system. In *Sumalia daraxa* (Figure 8b), the discal spot is present as a pair of parallel bands that span the compartments M_2_, M_1_, and Rs, which seem to be superimposed on the compressed band of the central symmetry system (BC). This BC is probably a fusion of the dBC and pBC. Proximal to these bands, a pair of bands of the basal symmetry system (dBB and pBB) (or DII) is present.

In *Tarattia lysanias* (Figure 8c), the central symmetry system has not only a pair of parallel bands (dBC and pBC) but also the central band (cBC) in the compartment Sc+R_1_ as a bar-like entity. A similar structure is also present in the proximal side of the potential DS area, which is likely the discal spot. These identifications of the central symmetry system are probably correct, because the intrasystem background (space between the dBC and pBC) is colored yellow, which is in parallel with the yellow intrasystem background in the border symmetry system on the same wing surface. Alternative identifications are a band of the central symmetry system and a pair of bands of the basal symmetry system. These alternative identifications are similar to those of the previous species, *Sumalia daraxa*. Either way, the identification of the discal spot is unequivocal.

Similar to the previous species, in *Dynamine aerate* (Figure 8d), the discal spot is present as a spot of bluish structural color. Similar bluish spots are also present anteriorly and posteriorly. In these two species (*Tarattia lysanias* and *Dynamine aerate*), because the spots are located in different compartments and because they are not connected, a reasonable interpretation when no additional information is available is that the central one is the discal spot, which is located in the potential DS area, and the anterior and posterior ones are the cBC, which is outside the potential DS area.

This interpretation is supported by another species, *Dynamine postverta* (Figure 8e). In this species, the discal spot is clearer than its anterior and posterior bands, which are likely the cBC. This discal spot is located at the conventional position in the potential DS area, confirming that this discal spot identification (and its related identifications of the cBC in this species and the identifications in the previous species) is correct.

In *Ragadia luzonia* (Figure 8f), the central symmetry system is expressed as a bold band. The identification of this band is tentative, and it is based on the observation that this bold band spans the potential DS area, whereas the band proximal to this band is far away from the potential DS area. The discal spot may be embedded within this bold band, and the discal spot is not expressed as a distinct entity. The identification of the basal symmetry system is less certain because the single band identified here as the central symmetry system may be simply the dBC, and the band identified here as the basal symmetry system may be the pBC, which are shown as alternative identifications in the figure.

### 3.7. The Morpho Paradox: The Discal Spot or cBC?

**Figure 9:**
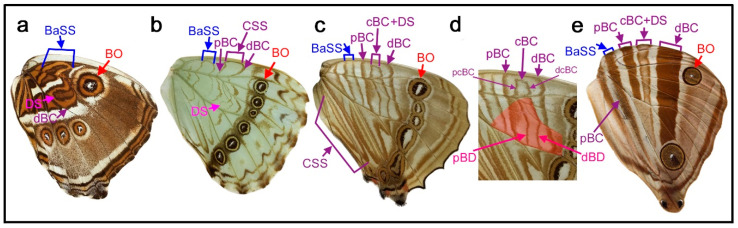
The discal spot and the central and basal symmetry systems of the genera *Morpho* and *Amathusia*. See Figure 1 for abbreviations. (**a**) *Morpho hecuba.* (**b**) *Morpho catenarius*. (**c**) *Morpho sulkowski*. (**d**) The discal area of the hindwing shown in c. The potential DS area is highlighted in red. (**e**) *Amathusia phidippus*.

The cases in the previous section (Section 3.6) highlighted a difficulty in distinguishing between the discal spot and the central band of the central symmetry system (cBC); in a few cases, the discal spot was identified based only on its location within the potential DS area because no other information was available. These characteristics are in contrast to those in the previous cases (Section 3.3), in which the discal spot and cBC are clearly distinguishable based on their structure and location. Additionally, in those cases, information from homology analysis among related species greatly helped identification of the discal spot. Important examples related to this issue are found in the genus *Morpho*. In both *Morpho hecuba* (Figure 9a) and *Morpho catenarius* (Figure 9b), the discal spot with typical morphology is present in the potential DS area together with the distal and proximal bands of the central symmetry system (dBC and pBC), although the color contrast is different between these two species. 

In *Morpho sulkowski* (Figure 9c,d), the dBC and pBC are clearly present, between which is an elongated element that covers anteriorly up to the compartment Sc+R_1_. Similarly, *Amathusia phidippus* (Figure 9e) has an elongated element that covers anteriorly up to the compartment C, the most anterior one, and posteriorly to the compartment CuA_1_. These presentations are rare cases in nymphalid butterflies. In these two species, the elongated element spans the potential DS area. Thus, they may be considered either the discal spot or cBC. Alternatively, they may be a fusion of the two. Importantly, the elongated element is a discal spot of Schwanwitsch’s type in structure, size, and location. The precise distinction among these three possibilities is not possible at this point. This indecisive state may be called the *Morpho* paradox.

### 3.8. Relationship between the Discal Spot and cBC

**Figure 10:**
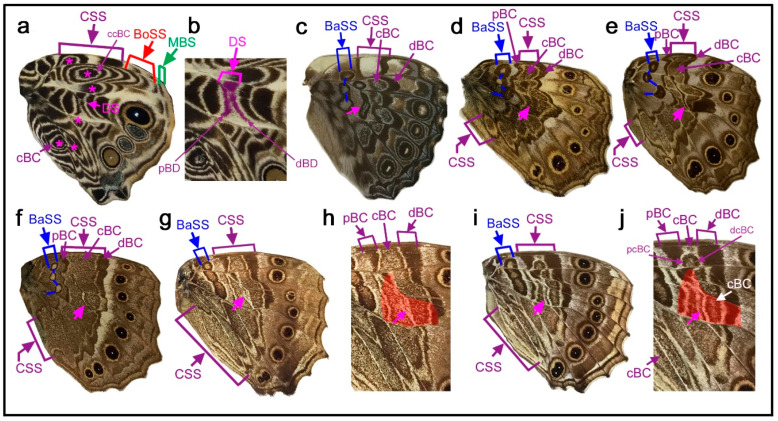
Relationship between the discal spot and the central band of the central symmetry system (cBC). Pink arrows without annotation indicate the discal spot. See Figure 1 for abbreviations. (**a**) *Smyrna blomfildia*. Asterisks indicate the center of the cBC (ccBC) in each compartment. (**b**) The discal area of the hindwing shown in a. The discal spot (DS) is composed of a dBD and pBD pair (colored in pink). (**c**) *Hamadryas feronia*. (**d**) *Neope niphonica*. (**e**) *Neope bremeri*. (**f**) *Neope muirheadi*. (**g**) *Neope yama*. (**h**) The discal area of the hindwing shown in g. The potential DS area is highlighted in red. (**i**) Another individual of *Neope yama*. (**j**) The discal area of the hindwing shown in i. The potential DS area is highlighted in red. Note that the structure of the cBC in this individual is similar to the elongated structure at the center of the central symmetry system in *Morpho sulkowski* (Figure 9c,d) and in *Amathusia phidippus* (Figure 9e).

To understand the central band of the central symmetry system (cBC) and its relationship with the discal spot, a case was examined in which the discal spot and cBC are clearly present: *Smyrna blomfildia* (Figure 10a,b). In this species, the discal spot is easily identifiable because a pair of parallel bands (dBD and pBD) are present on both sides of the discal cross vein. Anterior and posterior to the discal spot, there are multiple crescent bands with a core disk located in each compartment. The core disk corresponds to the cBC (and it may be the central band of the cBC, i.e., ccBC), and some of the multiple crescent bands at the distal and proximal sides of the circular core element correspond to the dBC and pBC, respectively (and some of them may be the distal and proximal bands of the cBC, i.e., dcBC and pcBC). This case confirmed that there are organizers of the central symmetry system at each compartment, similar to the border symmetry system, and that the discal spot is different from the cBC.

In *Hamadryas feronia* (Figure 10c), the central symmetry system is composed of serial eyespot-like structures, which are the cBC. The discal spot is an elongated oval on the discal cross vein and its anterior veins. The most anterior part is an isolated spot in the compartment Rs, which also belongs to the discal spot, as in *Agrias beatifica* (Figure 5h) and *Agrias claudina* (Figure 5i). Interestingly, the discal spot is colored blue inside, as are other eyespot-like structures of the central symmetry system, suggesting that the discal spot and the central symmetry system can be expressed in a morphologically similar fashion.

In the previous two species, the central symmetry system is completely divided by the wing veins, but in the genus *Neope*, it is only partially divided by the wing veins and forms a bold band that spans from the anterior to posterior margins. In *Neope goschkevitschii* (Figure 3e) and *Neope niphonica* (Figure 10d), cBC spots are still somewhat disk-like, although not completely, in each compartment, similar to a core disk of an eyespot. In contrast, in *Neope bremeri* (Figure 10e) and *Neope muirheadi* (Figure 10f), cBC spots are all fused completely to form a relatively smooth bold band from the anterior to posterior margins. An intermediate configuration between the disk-like and bold-band cBC is present in an individual of *Neope yama* (Figure 10g,h): the posterior portion is disk-like, and the anterior portion is bold band-like. Impressively, in another individual of *Neope yama* (Figure 10i,j), the cBC in the posterior portion of the wing is disk-like, but in the anterior portion of the same wing, the cBC is much narrower, forming an elongated band that resembles that of *Morpho sulkowski* (Figure 9c,d). This difference between the anterior and posterior portions of the cBC in the same wing of *Neope yama* (Figure 10i,j) is also present in *Morpho sulkowski* (Figure 9c,d). Importantly, discal spots distinctly exist in *Neope yama* (Figure 10i,j) in addition to the cBC, but no such element distinct from the cBC exists in *Morpho sulkowski* (Figure 9c,d).

In summary, the discal spot and cBC are clearly distinguishable in *Neope yama* (Figure 10i,j) and other species presented in this section. Based on these results, a tentative (and incorrect) conclusion is that the elongated element of *Morpho sulkowski* (Figure 9c,d) could be the cBC, and the discal spot might not be expressed in these species. However, this conclusion is inconsistent with the fact that other *Morpho* species (Figure 8a,b) have conventional discal spots.

A problem in interpreting the case of *Neope yama* (Figure 10i,j) may be that its discal spot is the simplest narrow band. If the discal spot is expressed as a pair of parallel bands (dBD and pBD) or more complicated structures, it is possible that both the discal spot and cBC could show elemental structures similar to each other, as seen in *Antanartia* and *Vanessa* (Section 3.3). Thus, they could easily fuse together and become indistinguishable. This interpretation is preferred to resolve the *Morpho* paradox. That is, the elongated element in *Morpho sulkowski* (Figure 9c,d) is a fusion between the discal spot (in the potential DS area) and cBC (outside the potential DS area). A close examination of this individual of *Morpho sulkowski* (Figure 9d) revealed that the cBC does not completely fuse with the discal spot.

### 3.9. Disappearance and Compromise

**Figure 11:**
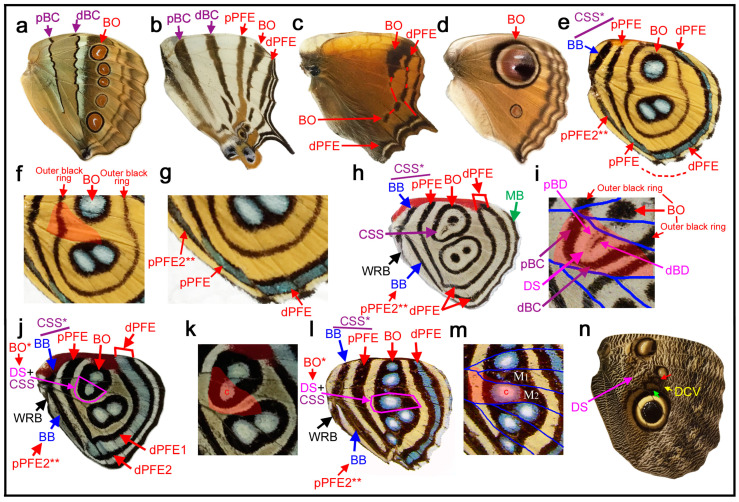
Disappearance and compromise of the discal spot. See Figure 1 for abbreviations. Alternative identifications are indicated by asterisks. Additional possible identifications are indicated by double asterisks. (**a**) *Stichophthalma camadeva*. (**b**) *Cyrestis camillus*. (**c**) *Hypanartia lethe*. Posterior border ocelli (BO) of the border symmetry system are expressed as simple black spots and aligned with the displaced distal parafocal element (dPFE) (each connected with red lines). These identifications were confirmed by the ventral color pattern (not shown). (**d**) *Junonia almana*. (**e**) *Callicore eunomia*. Fusion of the proximal parafocal element (pPFE) and dPFE is indicated by a broken line. See text for alternative identifications. (**f**) The discal area of the hindwing shown in e. The potential DS area defined by the wing veins is highlighted in red. (**g**) The posterior area of the hindwing shown in e. Bluish structural color is seen in the dPFE, pPFE, and pPFE2. (**h**) *Diaethria neglecta*. See text for alternative identifications. (**i**) The discal area of the hindwing shown in h. Small and weak bands (dBD and pBD) are enclosed by the dBC and pBC, which seamlessly fuse with the outer black ring of an eyespot (BO). The potential DS area defined by the wing veins is highlighted in red. (**j**) *Catacore kolyma*. The eyespot-like structure in the potential DS area is unlikely to be BO but likely to be a complex of the discal spot (DS) and the central symmetry system (CSS), based on homologies with the previous species. In this species, the dPFE is doubled, which is assured because the area between these two bands has a bluish structural color, a typical indication of the PFE. The pPFE may also be doubled. See text for alternative identifications. (**k**) The discal area of the hindwing shown in j. The letter “c” indicates an eyespot-like structure located in the distal portion of the potential DS area. The potential DS area defined by wing veins is highlighted in red. (**l**) *Callicore hesperis*. The eyespot-like structure in the potential DS area is unlikely to be BO but likely to be a complex of the DS and the CSS. See text for alternative identifications. (**m**) The discal area of the hindwing shown in l. The letter “c” indicates an eyespot-like structure located in the distal portion of the potential DS area. This eyespot-like structure is unlikely to be BO but a complex of the DS and the CSS. The compartments M_1_ and M_2_ are indicated. The potential DS area defined by the wing veins is highlighted in red. (**n**) *Caligo teucer* (female). The discal spot is located in the proximal portion of the potential DS area. A red arrow indicates an eyespot-like structure (BO) located in the distal portion of the potential DS area. A yellow arrow indicates the discal cross vein (DCV). A green arrow indicates the outermost rings of the major eyespot in the distal portion of the potential DS area.

Some species, such as *Stichophthalma camadeva* (Figure 11a) and *Cyrestis camillus* (Figure 11b), lack the discal spot despite the expression of the central symmetry system. In *Hypanartia lethe* (Figure 11c) and *Junonia almana* (Figure 11d), both the discal spot and the central symmetry system (as well as the basal symmetry system) are not present. In the latter species, the anterior eyespot is so large that a portion of the eyespot invades a portion of the potential DS area. The disappearance of the discal spot is likely more frequent in the hindwings than in the forewings, as shown in a previous study [19]. In fact, these four species have the discal spot in their forewings (not shown).

In *Callicore eunomia* (Figure 11e–g), the potential DS area is surrounded by eyespots (border ocelli; BO), and the discal spot is not expressed; indeed, most of the wing is occupied by the border symmetry system. However, in this species, the potential DS area is kept blank as an intrasystem background except for a band of an outer black ring of BO. Most species of the genus *Callicore* and its related genera appear to follow this rule. Identifications of proximal bands in this species (more generally in *Callicore* and its related genera) are difficult due to lack of specifying information. Schwanwitsch (1956) [18] identified a pair of the most proximal bands as a dislocated central symmetry system (CSS) (see alternative identification in Figure 11e). This identification is based on a proximal shift of the entire color patterns (compare Figure 11e with Figure 11a, for example). However, it is a rule that a pair of bands of the central symmetry system must sandwich the potential DS area. As seen in *Marpesia furcula* (Figure 3c), the CSS dislocation requires rearrangement of the wing veins, because the potential DS area is defined by the wing veins. If this rule is observed, the dislocation of the entire color patterns including the CSS in *Callicore* and its related genera without rearrangement of the wing veins cannot be accepted. If Schwanwitsch (1956) [18] is correct, this means that the potential DS area itself is dislocated proximally without rearrangement of the wing veins, which is unlikely to be true. Thus, one of the proximal bands is tentatively identified as the pPFE. The tentative pPFE has metallic blue coloration posteriorly, which is a feature of the PFE in general, and this band is directly connected with the dPFE at the tornus (Figure 11g), suggesting their circular nature. Moreover, the large BO in this species suggest a high expression of the border symmetry system including the pPFE. Thus, in the present study, the band of the pPFE was identified as such. The most basal band is less certain, but it was tentatively identified as the band of the basal symmetry system simply because of the lack of the central symmetry system. However, there is a possibility that this is also a part of the pPFE (doubled pPFE) (see below).

In the related species *Diaethria neglecta* (Figure 11h,i), the discal spot is present as a compromised form in the potential DS area. It is vanishingly small, but there are both distal and proximal bands (dBD and pBD). Importantly, this discal spot is surrounded by the distal and proximal bands of the central symmetry system (dBC and pBC), which form a half circle. These bands are smoothly connected with an outer black ring of the anterior eyespot. In this way, the distinction between the discal spot and eyespot (BO) was reasonably made, but the seamless fusion of the BC with an eyespot (BO) makes it difficult to notice their difference. Again, according to Schwanwitsch (1956) [18], a pair of proximal bands was annotated as the bands of the central symmetry system (see alternative identification in Figure 11h). However, in this species, the bands of the central symmetry system are already identified differently that surround the discal spot; additional bands of the central symmetry system (especially in the same compartment) cannot be permitted. Thus, likely identifications in the present study are the pPFE and BB (the band of the basal symmetry system). Considering that BO are very large in these species, their associated pPFE may be expressed well. The BB identification is less certain. This band could be another pPFE (see below).

Here, in *Catacore kolyma* (Figure 11j,k), the potential DS area is occupied by an eyespot-like structure, but based on homologies with the previous species, this eyespot-like structure in the potential DS area is probably not BO but the discal spot complexed with the central symmetry system (CSS). Interestingly, the dPFE is a bold band within which a bluish structural color exists. This bold band could be considered two bands. Extension of this structure to the proximal side may produce a pair of pPFE bands.

In *Callicore hesperis* (Figure 11l,m), a distal portion of the potential DS area is occupied by one of the eyespot-like structures. This is an exceptional case in *Callicore*. If this eyespot is BO, then the discal spot is not expressed, and the central symmetry system is not expressed, either. In that case, a possible identification is that the bands of the central symmetry system are dislocated proximally (see alternative identification in Figure 11l). However, the dislocated bands of the central symmetry system may be too far away from the potential DS area to be identified as such. A different (and probably correct) identification is that the eyespot-like structure in the potential DS area in this species is not BO but the discal spot itself complexed with the central symmetry system. If so, portions of the black bands that surround this discal spot may be considered the central symmetry system, which fuse with the bands of the border symmetry system, just like the previous species, *Diaethria neglecta* (Figure 11h,i) and *Catacore kolyma* (Figure 11j,k).

The genus *Caligo* and its related genera probably have the largest eyespots in butterflies, but in most species of the genus, the eyespots are outside the potential DS area. However, in females of *Caligo teucer* (Figure 11n), the outer black and brown rings of the major eyespot invade a distal portion of the potential DS area. Moreover, an eyespot-like structure (BO) exists in the most distal portion of the discal cell. That is, a distal portion of the potential DS area is partly occupied by the BO, but a proximal portion of the potential DS area is still devoid of it, where the discal spot is indeed present, although it is weakly expressed, in this species.

## 4. Discussion

### 4.1. The Issue of the Discal Spot

In this study, the discal spot in the hindwings of nymphalid butterflies was characterized morphologically and spatially. The characterization was long overdue because identification of the discal spot in a wing is often the important first step to decode the wing-wide color pattern configuration. The current illustration of the nymphalid groundplan in Nijhout (1991) [5] indicates that the discal spot is located only on the discal cross vein as a simple spot. Although similar vein dependence is illustrated both in Schwanwitsch (1924) [3] and Süffert (1927) [4], these three versions are all different in the size and inner structures of the discal spot in the hindwing (Figure 2). This disagreement probably reflects the diversity of the discal spot. However, the discal spot is more diverse than these three versions of the groundplan may imply.

### 4.2. Morphological Diversity of the Discal Spot and cBC

Morphologically, the discal spot is often a simple spot on the discal cross vein or its proximity even when the discal cross vein does not exist, as seen in five species in Figure 3. This simplest type is illustrated in the nymphalid groundplan of Nijhout (1991) [5]. The discal spot may be doubled to have distal and proximal bands (dBD and pBD) as a pair of paracore elements, as seen in nine species in Figure 4, and may also have the central band (cBD) as a core element, as seen in *Euryphura achlys* (Figure 4d) and *Euthalia lubentina* (Figure 4e). This aspect is illustrated in the nymphalid groundplan of Süffert (1927) [4]. Furthermore, the discal spot may be fragmented into two or more spots, as seen in *Agrias beatifica* (Figure 5h), *Agrias claudina* (Figure 5i), *Cethosia cydippe* (Figure 6d,e), and *Cethosia biblis* (Figure 6f–k). Conversely, these spots may fuse to be a conspicuous singular entity, which is likely the case in *Antanartia schaeneia* (Figure 5a) and *Antanartia delius* (Figure 5b). The discal spot may also merge with bands of the central symmetry system, as seen in *Cethosia biblis* (Figure 6f–k), *Vagrans egista* (Figure 8a), and *Sumalia daraxa* (Figure 8b). When the discal spot fuses with the central band of the central symmetry system (cBC), they are morphologically indistinguishable, as seen in *Morpho sulkowski* (Figure 9c,d) and *Amathusia phidippus* (Figure 9e). A possible eyespot-like complex of the DS and the CSS is seen in *Catacore kolyma* (Figure 11j,k) and *Callicore hesperis* (Figure 11l,m).

Moreover, individual variation in the discal spot in *Cethosia biblis* (Figure 6f–k) illustrates the flexibility of this system. The discal spot of this species is likely a pair of parallel bands if fully expressed. In *Nymphalis vaualbum* (Figure 7a), *Polygonia c-album* (Figure 7b,c), *Hypna clytemnestra* (Figure 7d), and *Issoria lathonia* (Figure 7e), the discal spot has a distinct white structure. Interestingly, in the case of *Polygonia c-album* (Figure 7b,c), the discal spot has two forms that are located closely to each other: the conventional one at the anterior portion and the white crescent structure at the posterior portion. This presentation is a special case where a portion of the discal spot differentiated into a conspicuous spot in the course of the color pattern evolution.

In many cases, such as in *Antanartia schaeneia* (Figure 5a), *Antanartia delius* (Figure 5b), *Neope goschkevitschii* (Figure 3e), and their related species (Figure 5 and Figure 10), the central band of the central symmetry system (cBC) exists, and it is clearly different from the discal spot. In some cases, such as *Vanessa* (*Antanartia*) *dimorphica* (Figure 5c) and *Vanessa* (*Cynthia*) *kershawi* (Figure 5e), the discal spot is difficult to distinguish from the central and posterior bands of the central symmetry system (cBC and pBC) in shape and color. However, color pattern comparisons with related species made it possible to identify the discal spot even in these species. In *Vanessa* (*Cynthia*) *virginiensis* (Figure 5f) and *Vanessa atalanta* (Figure 5g), the cBC is clearly different from the discal spot. In these cases, the cBC is more variable than the discal spot, suggesting that they are different in identify. These cases also suggest the importance of the location of the discal spot for its identification. In *Tarattia lysanias* (Figure 8c), *Dynamine aerata* (Figure 8d), and *Dynamine postverta* (Figure 8e), the discal spot and cBC are morphologically similar, but because they are located differently without fusion, they can be identified as such.

### 4.3. The Morpho Paradox

Although *Morpho hecuba* (Figure 9a) and *Morpho catenarius* (Figure 9b) have conventional discal spots, *Morpho sulkowski* (Figure 9c,d) and *Amathusia phidippus* (Figure 9e) have the elongated element at the center of the central symmetry system. The analyses of *Neope* species could be interpreted such that the elongated element can be identified as the cBC. However, if it is the cBC, the typical discal spot in *Morpho hecuba* (Figure 9a) and *Morpho catenarius* (Figure 9b) becomes misidentified (the *Morpho* paradox). In contrast, the discal spot and cBC can be distinguished clearly in the cases of *Antanartia* and *Vanessa* (Figure 5).

The *Morpho* paradox was resolved by introducing a state of fusion. That is, the elongated structure is likely the fusion between the discal spot (in the potential DS area) and cBC (outside the potential DS area). This interpretation is consistent with the interpretation of *Tarattia lysanias* (Figure 8c), *Dynamine aerata* (Figure 8d), and *Dynamine postverta* (Figure 8e), in which the discal spot and cBC, with similar morphology, were distinguished based on their locations (Section 3.6). Therefore, this study concludes that the discal spot illustrated in the nymphalid groundplan of Schwanwitsch (1924) [3] is a fusion between the discal spot (in the potential DS area) and cBC (outside the potential DS area). The fusion of different elements to form a continuous band due to dislocations has been known as pierellization [5,23]. Since the fusion between the discal spot and cBC does not involve dislocations, such fusion may be produced without difficulty.

This issue of the discal spot in the nymphalid groundplan might have stemmed from the fact that previous studies [3,4,5,6,7] did not clearly recognize the cBC as an independent element, although Schwanwitsch (1924) [3] was careful enough to recognize the cBC as granulata (GI and GII) within the central symmetry system. Otaki (2012) [7] also incorrectly concluded that the discal spot is a core element of the central symmetry system. Although Nijhout (1991) [5] recognized eyespot-like structures of the cBC well in *Smyrna blomfildia* and other species, the cBC was considered not an element but just a field of the central symmetry system, rejecting the identification of granulata in Schwanwitsch (1924) [3].

The *Morpho* paradox was resolved as described above, but the paradox also raises another question about the possibility of an overlapping expression of two elements. In the discussion above, it was assumed that an element has only a single identification. Although this assumption is reasonable, two different elements may be expressed in an overlapping manner, at least theoretically, especially in the case of the discal spot and cBC because they can be expressed within the potential DS area without notable interference with each other. For example, in *Sumalia daraxa* (Figure 8b), the discal spot appears to be superimposed on the band of the central symmetry system. Importantly, cases of *Catacore kolyma* (Figure 11j,k), *Callicore hesperis* (Figure 11l,m), and *Diaethria neglecta* (Figure 11h,i) suggest that complex fusions of the discal spot and the central symmetry system (and the border symmetry system) in the potential DS area may be possible. Thus, there is a small possibility that the discal spot in *Morpho sulkowski* (Figure 9c,d) and *Amathusia phidippus* (Figure 9e) may indeed be a complete superimposition of the discal spot and cBC in the potential DS area. Related to this issue, it has been known that positional information for a black (dark) color and a light color may be expressed together, which is based on observations of overpainting during color pattern formation [30,31,32]. Elemental interactions, including fusion, repulsion, and superimposition, may be studied in the future.

### 4.4. The Potential DS Area

Spatially, the location of the discal spot is not always on the discal cross vein. The discal cross vein does not always exist, but the location of the discal spot appears to be unrelated to this fact. The location of the discal spot can be different between the dorsal and ventral sides of the same wing in *Cethosia hypsea* (Figure 6a–c), although the venation system is the same. Nonetheless, the location of the discal spot is largely limited within a given area defined by the wing veins. This area was termed the potential DS area (see Figure 1b), which is an area extension of Süffert’s type. Coincidentally, the discal spot of *Thaumantis odana* (Figure 5j) nicely corresponds to the potential DS area. The fact that the expression of the discal spot is mostly limited within this area may be called the DS area rule. Thus, one of the evolutionary strategies to change the relative location of elements in a wing is to change the relative location of the DS area by changing the whole structure of the wing veins, as seen in *Marpesia furcula* (Figure 3c). In *Callicore*, *Diaethria*, and *Catacore* (Figure 11e–m), the proximal bands that were identified as the dislocated central symmetry system (CSS) in Schwanwitsch (1956) [18] do not accompany the rearrangement of the wing veins, and this is the reason that these bands are unlikely to be the CSS.

Assuming that the DS area rule is correct, this rule can now be used to identify the discal spot and the central symmetry system that flanks the discal spot. For example, this rule confirms the identification of the discal spot in difficult cases, such as *Tarattia lysanias* (Figure 8c) and *Dynamine aerata* (Figure 8d). The bold band at the center of the wing in *Ragadia luzonia* (Figure 8e) can also be identified as the central symmetry system, although its proximal band is difficult to identify. In *Morpho sulkowski* (Figure 9c,d), the discal spot in the potential DS area likely forms a fusion with the cBC outside the potential DS area, as discussed above. Therefore, the DS area rule works well to identify the discal spot without significant exceptional cases.

The potential DS area is not completely occupied by elements of the border symmetry system. There are some exceptional cases in which the potential DS area is partially invaded by border ocelli, as seen in *Junonia almana* (Figure 11d) and *Caligo teucer* (Figure 11n). However, even in those cases, the potential DS area is not completely occupied; only its distal portion is occupied. Moreover, even when the potential DS area is completely enclosed (but not occupied) by the border symmetry system, the potential DS area is largely kept blank, as in *Callicore eunomia* (Figure 11e–g), or has the discal spot in a compromised fashion, as in *Diaethria neglecta* (Figure 11h,i). In *Catacore kolyma* (Figure 11j,k) and *Callicore hesperis* (Figure 11l,m), an eyespot in the potential DS area may be considered the discal spot itself complexed with the central symmetry system, as an extension of *Diaethria neglecta* (Figure 11h,i). Similarly, no case was found in this study in which the potential DS area is completely occupied by the basal symmetry system, although a case of partial occupation is seen in *Morpho sulkowski* (Figure 9c,d). Therefore, it is likely that at least some portion of the potential DS area is “saved” or “protected” for the discal spot from potential invasion of other elements. The discal spot may not be expressed at all, as seen in five species in Figure 11, and in some cases, the discal spot and the central symmetry system together are not expressed at all, even when the border symmetry system is well expressed, as seen in three species in Figure 11. More precisely, the expression level (i.e., size and elaboration) of the discal spot may be positively correlated with the expression level of the central symmetry system. This positive correlation may be because the discal spot is nested within the central symmetry system and because they are a collaborating unit. In addition, the expression level of the discal spot may be negatively correlated with the expression level of the border symmetry system. This negative correlation may be because a distal portion of the potential DS area is partially occupied when eyespots are large or located relatively close to the potential DS area. In that case, the discal spot appears to be relegated to the proximal portion of the potential DS area. Elemental repulsion may play a role in this relegation process [33,34]. These phenotypic correlations among symmetry systems do not necessarily reflect expression patterns of *Wnt* family genes; *WntA* is expressed in the central symmetry system but not in the discal and border symmetry systems [19,20,21].

### 4.5. The Discal Symmetry System As an Independent Symmetry System

The discal spot is considered an independent symmetry system despite its miniature nature because it has its own distal, proximal, and central bands (dBD, pBD, and cBD), as other symmetry systems do. The discal “spot” is often not a simple spot; the discal “spot” (singular) often has two bands or even a few spots (plural). Thus, the terminology discal “spot” is somewhat misleading. In other families of Lepidoptera, the discal spot forms a large and conspicuous eyespot-like structure, especially in Riodinidae and Saturniidae, as indicated in Nijhout (1991) [5]. In Nymphalidae, such a conspicuous eyespot-like structure is rare, but the discal spot of the ventral forewing in *Vanessa* (*Bassaris*) *gonerilla* shows a circular pattern [7]. The discal spot of the ventral hindwing of *Historis acheronta* is also circular (not shown). Importantly, *Catacore kolyma* (Figure 11j,k) and *Callicore hesperis* (Figure 11l,m) may be considered to have circular eyespot-like discal spots, although they may be fusion products of the discal spot and the central symmetry system. More comprehensive surveys may find additional examples of circular discal spots in Nymphalidae. Considering the diversity of the discal spot, it may be called the discal symmetry system more appropriately. A possible illustration of the discal symmetry system based on the present study may be just like the illustration of Süffert (1927) [4] (Figure 2b). Together with the recognition of the cBC in Schwanwitsch (1924) [3], this study presented a more accurate understanding of the discal and central symmetry systems.

The discal symmetry system is different from other symmetry systems in the following points. First, the discal symmetry system is the only system that may be placed on the wing vein, although this is not always the case. No other systems or elements are placed directly on the wing vein in the nymphalid groundplan. Second, related to the first point, the location of the discal symmetry system is mostly limited within the potential DS area that is defined by the wing veins. As a result, the discal symmetry system does not span from the anterior to posterior margins but is limited in and near the potential DS area. In contrast, the three major symmetry systems all potentially form a band or a spot array that spans from the anterior to posterior margins, although a possible exception is the basal symmetry system (which requires additional studies). Third, in some species, the central symmetry system in each wing compartment can have an independent core disk (i.e., cBC), similar to that of the eyespot. That is, the cBC and the discal symmetry system are readily distinguishable in these cases, indicating that the discal symmetry system is independent of the central symmetry system. Fourth, the nesting configuration within a different and larger symmetry system is unique in the discal symmetry system.

Additionally, according to a molecular study, *wingless* and its related genes *Wnt6* and *Wnt10* are expressed in the prospective discal spot, suggesting their role in the expression of the discal spot [19,20]. Moreover, *WntA* functions to express the central symmetry system but not the discal symmetry system, demonstrating that these two systems are different at the molecular level [21]. These genes can be used as expression markers for symmetry systems to validate the distinction between the discal symmetry system and the central symmetry system presented in this study, considering that these two systems are often difficult to distinguish just based on morphological analyses (e.g., the *Morpho* paradox). Interactions between these two systems may also be validated; the *WntA* knockout revealed an unclear or “hidden” discal spot due to the elimination of the central symmetry system [21].

### 4.6. Development and Morphological Similarity

Considering that the potential DS area is determined by the wing veins (and thus by lacunae) at the early stage of development, the potential DS area may have a special function in determining the overall wing color patterns during development. As a result, the discal symmetry system is likely determined prior to other symmetry systems. This hypothesis is consistent with the fact that the discal symmetry system is colored first during adult wing development at the pupal stage in some nymphalid butterflies [31]. Furthermore, the discal symmetry system is known to be widespread among families of Lepidoptera [19] despite its relatively inconspicuous nature, suggesting its importance not in visual communication but in determining wing-wide color patterns during development and evolution.

Notably, the discal symmetry system morphologically sometimes resembles that of the central symmetry system itself, as seen in *Cymothoe egesta* (Figure 4f,g) and *Hamadryas feronia* (Figure 10c); the discal symmetry system is similar to a miniature version of the central symmetry system. The discal symmetry system and cBC (and pBC) are structurally similar and often merge to the point that their distinction is difficult, as discussed above. These cases suggest that the discal and central symmetry systems could be morphologically similar, although in most species, these two systems are morphologically different and they are indeed different at the molecular level [21]. This could be a self-similar relationship, in which a part is contained in the whole, but a part is considered independent of the whole. Even if this self-similarity hypothesis is not correct, the central symmetry system can be defined as the system that flanks the discal symmetry system. This may be called the discal-central positioning rule. This rule, together with the DS area rule, restricts the location and configuration of both the discal and central symmetry systems within a given wing of nymphalid butterflies.

The findings in this study can be understood as a serial induction process of organizers. As soon as the lacunae of the venation system are established in the larval imaginal disk during development, the primary organizer for the discal symmetry system is induced in the potential DS area. This organizer then induces the secondary organizer at the center of the central symmetry system. The secondary organizer then induces the tertiary organizers for the distal and proximal bands of the central symmetry system (dBC and pBC). In the case of a lycaenid butterfly, *Zizeeria maha*, the distance between the discal spot and the arrays of spots of the central symmetry system that surround the discal spot is genetically controlled during development, according to a mutagenesis study [35,36]. The distance may be dependent on the activity of the organizer that releases morphogenic signals for positional information for the spots of the central symmetry system.

It has been known that the eyespot (BO) and its corresponding parafocal element (PFE) are self-similar, which is known as the self-similarity rule [8]. In this case, the PFE (i.e., paracore element) is a part of the corresponding BO (i.e., core element). Thus, they are collectively called the border symmetry system. The potential self-similarity of the discal and central symmetry systems may be considered an application of the self-similarity rule proposed for the border symmetry system. The distortion hypothesis and its associated induction model [8,13,14,15,32,37,38,39,40,41] may be able to conceptually explain how this self-similar configuration is produced from a serial induction process.

## 5. Conclusions

This study demonstrated the diversity of the discal spot as an independent miniature symmetry system. In this sense, the discal spot may be called the discal symmetry system. This study also showed that the discal symmetry system is located in the potential DS area or its proximity. In these two points, Süffert’s type is more appropriate than Schwanwitsch’s and Nijhout’s types. In addition, this study clearly showed that the discal symmetry system differs from the central band of the central symmetry system (cBC), which is occasionally elaborated but has not been recognized well in the literature. These two different structures nonetheless may fuse together, which often confuses elemental identifications. Overall, the present study revised the current nymphalid groundplan in minor but important points and deepened the understanding of the making of butterfly wing color patterns.
